# First Complete Genome Sequence of *Salmonella enterica* subsp. *enterica* Serovar Typhimurium Strain ATCC 13311 (NCTC 74), a Reference Strain of Multidrug Resistance, as Achieved by Use of PacBio Single-Molecule Real-Time Technology

**DOI:** 10.1128/genomeA.00986-14

**Published:** 2014-10-02

**Authors:** Yasunobu Terabayashi, Ayaka Juan, Hinako Tamotsu, Noriko Ashimine, Kazuma Nakano, Makiko Shimoji, Akino Shiroma, Kuniko Teruya, Kazuhito Satou, Takashi Hirano

**Affiliations:** Okinawa Institute of Advanced Sciences, Uruma, Japan

## Abstract

We report the first complete genomic sequence of *Salmonella enterica* subsp. *enterica* serovar Typhimurium strain ATCC 13311, the leading food-borne pathogen and a reference strain used in drug resistance studies. *De novo* assembly with PacBio sequencing completed its chromosome and one plasmid. They will accelerate the investigation into multidrug resistance in *Salmonella* Typhimurium.

## GENOME ANNOUNCEMENT

*Salmonella enterica* subsp. *enterica* serovar Typhimurium (*S.* Typhimurium) strain ATCC 13311 (NCTC 74), the type strain of *S.* Typhimurium, was originally isolated in 1911 from mutton by Castellani and Chalmer (http://www.phe-culturecollections.org.uk/products/bacteria/detail.jsp?refId=NCTC 74&collection=nctc) (1, 2). *Salmonella*, the leading bacterial food-borne pathogen, encompasses a large group divided into 6 subspecies and more than 2,500 serovars (3, 4). *S.* Typhimurium causes human gastroenteritis and mouse typhoid (5). Its multidrug-resistant strains have spread worldwide (6). In Japan, 47 to 189 strains were isolated from patients every year from 2000 to 2011 (http://thor.dfvf.dk/portal/page?_pageid=53,1&_dad=portal&_schema=PORTAL), including multidrug-resistant ones (7). PCR-based studies revealed the association between genomic mutations in the quinolone resistance-determining region and naldixic acid resistance (8, 9). *S.* Typhimurium ATCC 13311, a quinolone-susceptible strain, has been used as a reference in multidrug resistance studies (10). Despite the importance of exhaustive analysis, its complete genomic sequence has not been reported.

Genomic information leads to rapid and accurate diagnostics, including serotyping and detection of drug resistance (11). Whole-genome sequencing provides fine resolution for outbreak analysis and short-term epidemiology (12). However, the *Salmonella* genome is difficult to determine using only short-read sequencers because of the long identical loci. For example, *S.* Typhimurium LT2 contains 2 pairs of identical regions spanning over 5 kb, coming from a Gifsy prophage and a cytochrome *c* gene cluster, respectively (13). Furthermore, sizes of plasmids vary among strains (14). The sequence of plasmids associated with drug resistance is equally important as that of chromosomes (15).

Here, we report the first complete sequence of *S.* Typhimurium ATCC 13311 obtained by using single-molecule real-time sequencing (16). We already demonstrated the benefits of its long read and equal coverage by determining the complete sequence of Okinawan *Helicobacter pylori* (17).

The genomic DNA obtained from ATCC was purified using the MOBIO PowerClean DNA CleanUp kit, followed by 20-kb library construction for P5-C3 chemistry without shearing. Aliquots of libraries were sequenced after BluePippin size selection at 7 kb to obtain long libraries crossing over identical sequences. The remaining libraries were run without size selection to acquire plasmid information in short fragments. Eight SMRT cells of size-selected and nonselected libraries each (16 cells total) were sequenced by PacBio RS II with a 180-min movie. Maximum/average subread lengths were 26,443/1,798 bp and 25,604/5,706 bp, respectively. *De novo* assembly was conducted by using a hierarchical genome assembly process 2 (HGAP2) workflow, including Quiver consensus polishing, followed by Minimus2 and Quiver again (18). Two circular contigs were obtained, one representing a chromosome (4,793,299 bp, 52.2% G+C content) and the other, a plasmid (38,457 bp, 40.7% G+C content). An identical pair of 5,420-bp cytochrome *c* gene clusters on the chromosome was successfully captured from PacBio long reads of BluePippin size-selected libraries.

This complete genomic sequence of *S.* Typhimurium ATCC 13311 will accelerate investigations into multidrug resistance.

### Nucleotide sequence accession numbers.

Complete genome sequences of *Salmonella* Typhimurium ATCC 13311 chromosome and plasmid were deposited in DDBJ/EMBL/GenBank under the accession numbers CP009102 and CP009103, respectively. The methylation motifs, determined by kinetic analysis, were deposited in REBASE (http://tools.neb.com/~vincze/genomes/view.php?enzname=M.Sen13311DamP) (19).
